# Student perceptions of the impact of quality matters essential standards in an animal physiology course

**DOI:** 10.1093/tas/txad112

**Published:** 2023-09-21

**Authors:** Kylie Deuser, Rebecca P Sanchez, Arlene Mendoza-Moran, Bethanne Winzeler, Yaxin Zheng, Shweta Trivedi

**Affiliations:** Animal Science Department, North Carolina State University, Raleigh, NC 27695; Digital Education and Learning Technology Applications (DELTA), North Carolina State University, Raleigh, NC 27695; Digital Education and Learning Technology Applications (DELTA), North Carolina State University, Raleigh, NC 27695; Digital Education and Learning Technology Applications (DELTA), North Carolina State University, Raleigh, NC 27695; Statistics Department, North Carolina State University, Raleigh, NC 27695; Animal Science Department, North Carolina State University, Raleigh, NC 27695

**Keywords:** online course design, online course quality, online education, quality matters, quality assurance, student perception

## Abstract

As online learning becomes increasingly popular in higher education, the quality of courses that utilize this modality is becoming a focus of inquiry. Quality Matters (**QM**) is a leading quality assurance organization that reviews online and hybrid (partially online, partially in-person) courses for standards of pedagogy and instructional design and certifies courses that sufficiently meet these standards. In this study, we examine student perceptions of course quality in a hybrid three-credit-hour animal science course that has been certified by QM. The class met twice a week for 1.25 h with one class period online and one in person. It consisted of 11 modules, each of which included learning content, learning activities, and assessments. Upon completion, 46 of 114 students completed a survey in which they rated the course on each of the 21 QM essential standards (Fifth edition). Descriptive analysis revealed that for 19 of the 21 specific review standards, 75% to 91% of students agreed or strongly agreed that the course reflected the best practice described in the standard. For the other two standards, over half of students (72%, 63%) agreed or strongly agreed that best practices were reflected in course design. Another way to examine the data is to collapse specific review standards into eight general review categories as specified by QM; the collapsed data revealed that 75% to 88% of students agreed or strongly agreed that the course design reflected the eight general course design standards. The percentage of students disagreeing that the course reflected each best practice was 11% or lower. Cronbach analysis to examine the internal consistency of the QM questionnaire (0.96), indicated instrument reliability and stability. A principal component analysis of the data conducted to further examine features and patterns of student responses revealed four primary factors that students rated highly (learning objectives, learner interaction and engagement, accessibility and usability, and clarity) that explained 78% of the data variance. This study demonstrates that the high quality of course design and delivery in a QM-certified course is clear to students. and provides justification for the investment in high-quality online and hybrid course design. In the future, we plan to compare student perceptions of course quality in a course that has not been QM-certified with one that has, as well as the impact of those revisions on student outcomes.

## INTRODUCTION

Online learning is becoming increasingly prevalent at colleges and universities (He, Xu, & Kruck, 2014; Guppy, Verpoorten, Boud, Lin, Tai, & Bartolic, 2022). Students in higher education are open to learning online (Bali & Liu, 2018) and recognize that instructional design is a critical component of successful online courses (Nassoura, 2020). To ensure the instructional design quality of their online courses, higher education institutions across the nation have begun to implement quality assurance efforts such as the Quality Matters (**QM**) Quality Assurance System. QM is a not-for-profit organization that has gained national acknowledgement. This system consists of a process for the design and review of online or hybrid (i.e., partially online) courses based on a rubric that comprises 42 specific review standards subsumed under eight overarching general review standards (in the fifth edition for higher education). General Standard (**GS**) 1 focuses on providing clarity in the course’s overall design, establishing expectations for students (e.g., prerequisite knowledge, technology requirements, computer skills), and providing guidance to help them be successful in the course. GS 2 establishes standards for clear, specific, measurable, level-appropriate learning objectives (i.e., what students should be able to do upon completion of the course). GS 3 addresses the need for course assessments to evaluate students’ progress toward and achievement of stated learning objectives. GS 4 supports the inclusion of instructional materials that enable students to achieve the learning objectives. GS 5 addresses the need to facilitate and support student engagement and interaction with the instructor, materials, and classmates. GS 6 covers the need for course technologies to support student achievement of the learning objectives. GS 7 ensures that the course facilitates student access to available support (academic, technical, accessibility, and student services). GS 8 addresses ways to reflect a commitment to making the course and its components accessible and usable for all students. Taken together, the eight QM general standards provide a framework for applying best practices in online course design with the goal of facilitating student success.

Courses can be submitted for official peer review by the QM organization. Course review can result in certification by QM if standards are sufficiently met (Shattuck et al. 2014). The soundness of the QM rubric was examined by being compared to a set of standards put forth by the Council for Higher Education Accreditation and the eight regional accrediting agencies. The QM rubric was determined to be congruent with the published accreditation standards for online education (Sadaf et al. 2019).

The legitimacy of the QM rubric having been established; the next step is to begin to document the impact of the approach on students. Impact can be measured in multiple ways; one important consideration is student perception of course quality, which directly affects engagement with online learning (Baloran et al. 2021). Student engagement with online learning is the “extent to which students actively engage by thinking, talking, and interacting with the content of a course, the other students in the course, and the instructor” (Dixson 2015). Student perceptions could also be used to revise current QM standards in future additions of the rubric (Shattuck 2015). Although the QM rubric was created to reflect best practices in the instructional design of online courses, there is little information about whether students recognize these best practices. The purpose of this study was to gauge student perceptions of course quality in a QM-certified course in order to better understand the learners’ perspective and determine potential avenues for continuous course improvement.

## MATERIALS AND METHODS

### Course Description and QM Review

Physiology of Domestic Animals is a three-credit-hour course on mammalian physiology taught in the fall and spring semesters at North Carolina State University (**NC** State). It is a content-heavy required core course for animal science students, with a high enrollment of 110 to 140 participants each semester. It utilizes Moodle, a learning management system (**LMS**), to present course materials, activities, and assessments to track student progress. The course employs a hybrid design, with components being taught online and in person. It consists of 11 learning modules, each with a practice multiple choice question assignment and diagram labeling assignment. These assignments have three attempts before the deadline and accept the highest grade. There are also five quizzes and five congruent short answer assignments throughout the semester. The quizzes are timed, have one attempt, and consist of short answers, multiple choice, true/false, fill in the blank, and diagram labeling questions. This course was selected by the Digital Education and Learning Technology Applications (**DELTA**) unit at NC State for course design modifications and review of course quality to meet QM standards. The instructor attended face-to-face group meetings, individual consultations, participated in asynchronous online training and discussions, designed and applied improvements to an online course, submitted the course for internal review, and completed course modifications based on the peer reviews. The course was then submitted for the official QM certification process, which involves an official independent review by three outside trained peer reviewers.

For QM certification the peer reviewers assess whether the course meets each of the 42 specific review standards in the QM rubric (Fifth Edition) (QM Higher Education Rubric, Fifth Edition., 2018). In order to be certified, a course must meet 23 standards that are deemed essential for course quality and earn at least 85 out of 100 total possible points in the review. The Physiology of Domestic Animals course met these requirements and received QM certification in October 2021, indicating professional recognition that it met standards for high-quality course design.

### Participants and Procedures

Participants for this study involved students enrolled in the Physiology of Domestic Animals course in Spring 2022. The 114 students enrolled in the course were invited to complete a 25-item online Qualtrics survey (Supplementary Material) at the end of the course. Students were informed that participation was voluntary, the survey was not a graded assignment, and all responses would remain anonymous. There was a prize raffle incentive of eight NC State Bookstore t-shirts, four Tervis tumbler mugs, and two backpacks. The NC State IRB approved this research study (IRB 24779).

Of the 114 enrolled students, 46 completed the survey (40% response rate) and 39 included their demographic information. Among these students, 10.26% identified as male, and 89.74% identified as female. With respect to academic status of students, 10.26% were first-year students, 30.77% were sophomores, 51.28% were juniors, and 7.69% were seniors. Animal Science majors from the traditional 4-year track accounted for 79.49% of respondents, and 20.51% were transfer students.

### Instrumentation

Qualtrics software was used for the online survey platform tool. The link to the survey was distributed via email with a reminder email sent 3 days before the survey closed. After completing an informed consent to participate, students were able to access the survey, which consisted of 21 QM questions and 4 demographic questions. The QM questions represented the 21 essential specific review standards in the QM rubric, Fifth edition. These specific review standards can be grouped into overarching “general review standards,” each of which covers a broader concept in instructional design. The eight general review standards are Course Overview and Introduction (two of the specific review standards in our survey fall under this general standard), Learning Objectives (five items in our survey), Assessment and Measurement (three items), Instructional Materials (two items), Learner Interaction and Engagement (three items), Course Technology (two items), Learner Support (two items), and Accessibility and Usability (two items). The response option for each survey item was a Likert-type scale with responses ranging from 0 to 4 (0 = Strongly Disagree, 1 = Disagree, 2 = Neutral, 3 = Agree, and 4 = Strongly Agree).

### Data Analysis

The survey data were analyzed to determine student perceptions of course quality based on the instructional design standards of QM. Descriptive analysis (percentage, mean and standard deviation) was conducted for each item. The grand percentage was calculated for each of the eight general standards. Cronbach analysis was conducted to examine the internal consistency of the QM questionnaire.

To further examine the important features and patterns from student responses on QM standards, a multivariate data analysis using principal component analysis (**PCA**) was conducted to summarize students’ recognition of QM standards in their online learning experience. This method is conducted by transforming the 21 survey questions into a smaller set of variables called principal components which retain the most valuable features from the data. All analyses were performed in R (R Core Team, 2019) and RStudio (version 1.0.143). A Kaiser–Meyer–Olkin (**KMO**) analysis was made as a measure of sampling adequacy Kaiser (1960). The KMO values for the overall data are greater than 0.8 (KMO = 0.85), and the individual variables are at least larger than 0.75 which indicates our sampling is adequate.

## RESULTS

### Descriptive Results

The extent to which students agreed that the course reflected best practices in instructional design is shown in Table 1. Percentage, mean, and standard deviation are shown for each specific review standard (i.e., individual survey item), and grand percentage is noted for each general standard (i.e., overarching concept).

**Table 1.** Percentage, mean, and standard deviation of response options for QM standards impact on student learning

Items				Percentage			Mean	SD
	*n*	Strongly agree (4) (%)	Agree (3) (%)	Neutral (2) (%)	Disagree (1) (%)	Strongly disagree (0) (%)		
Standard 1. Course overview and introduction								
Q1 Clear instructions for getting started	46	52.20	32.60	10.90	4.30	0.00	3.33	0.84
Q2 Purpose and structure of the course	46	56.50	34.80	8.70	0	0	3.48	0.66
Grand percentage		54.3	33.7	9.8	2.2	0		
Standard 2. Learning objectives								
Q3 Measurable course learning perspectives	46	50.0	41.3	6.5	2.2	0	3.39	0.71
Q4 Learning objectives consistent with course-level objections	46	45.7	41.3	6.5	4.3	2.2	3.24	0.92
Q5 Learning objectives stated from learner’s perspectives	46	56.5	28.3	13	2.2	0	3.39	0.80
Q6 Clear relationship between learning objectives and course activities	46	47.8	37	13	2.2	0	3.30	0.79
Q7 Learning objectives suited to the level of the course	46	43.5	34.8	10.9	8.7	2.2	3.09	1.05
Grand percentage		48.7	36.5	10.0	3.9	0.9		
Standard 3. Assessment and measurement								
Q8 Assessments measure the learning objectives	46	32.6	50.0	15.2	2.2	0	3.13	0.75
Q9 Clearly stated course grading policy	46	45.7	34.8	17.4	0	2.2	3.22	0.89
Q10 Provided grading criteria tied to grading policy	46	28.3	34.8	28.3	6.5	2.2	2.80	1.00
Grand percentage		35.5	39.9	20.3	2.9	1.4		
Standard 4. Instructional materials								
Q11 Instructional materials consistent with learning objectives	46	41.3	41.3	10.9	4.3	2.2	3.15	0.94
Q12 Clearly explained use of learning materials	46	43.5	28.3	21.7	6.5	0	3.09	0.96
Grand percentage		42.4	34.8	16.3	5.4	1.1		
Standard 5. Learner interaction and engagement								
Q13 Learning activities consistent with objectives	46	34.8	43.5	15.2	6.5	0	3.07	0.88
Q14 Opportunities for learner interaction	46	34.8	47.8	15.2	2.2	0	3.15	0.76
Q15 Response time and feedback	46	30.4	47.8	21.7	0	0	3.09	0.72
Grand percentage		33.3	46.4	17.4	2.9	0		
Standard 6. Course technology								
Q16 Tools support the learning objectives	46	37	47.8	13	2.2	0	3.20	0.75
Q17 Technology tools to promote active learning	46	34.8	39.1	19.6	6.5	0	3.02	0.91
Grand percentage		35.9	43.5	16.3	4.3	0		
Standard 7. Learner support								
Q18 Instructions for technical support	46	39.1	39.1	19.6	2.2	0	3.15	0.82
Q19 Institution’s accessibility policies	46	43.5	32.6	23.9	0	0	3.24	0.82
Grand percentage		41.3	35.9	21.7	1.1	0		
Standard 8. Accessibility and usability								
Q20 Course navigation easy to use	46	45.7	34.8	17.4	2.2	0	3.24	0.82
Q21 Information about accessible technologies	46	43.5	41.3	15.2	0	0	3.28	0.72
Grand percentage		44.6	38	16.3	1.1	0		
Overall		42.2	38.7	15.4	3.1	0.5	3.19	0.84

**Figure 1.**
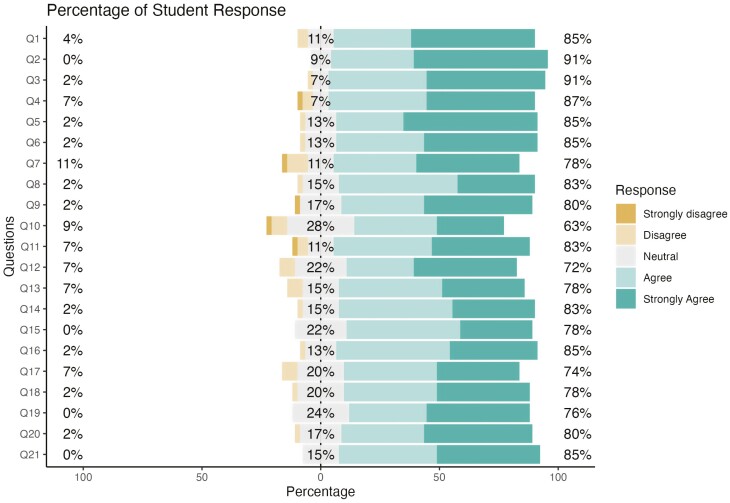
Percentage of student response. The percentage of student responses for each question in the survey is shown in accordance with a Likert-type scale ranging from strongly disagree to strongly agree.

General Standard 1, “Course Overview and Introduction,” had two questions and a grand percentage of 54.3% that strongly agree and 0% that strongly disagree. Standard 2, “Learning Objectives,” had five questions and a grand percentage of 48.7% that strongly agree and 0.9% that strongly disagree. Standard 3, “Assessment and Measurement,” had three questions and a grand percentage of 35.5% that strongly agree and 1.4% that strongly disagree. Standard 4, “Instructional Materials,” had two questions and a grand percentage of 42.4% that strongly agree and 1.1% that strongly disagree. Standard 5, “Learner Interaction and Engagement,” had three questions and a grand percentage of 33.3% that strongly agree and 0% that strongly disagree. Standard 6, “Course Technology,” had two questions and a grand percentage of 35.9% that strongly agree and 0% that strongly disagree. Standard 7, “Learner Support,” had two questions and a grand percentage of 35.9% that strongly agree and 0% that strongly disagree. Standard 8, “Accessibility and Usability,” had two questions and a grand percentage of 44.6% that strongly agree and 0% that strongly disagree. The overall percentages for all the standards were 42.2% strongly agree, 38.7% agree, 15.4% neutral, 3.1% disagree, and 0.5% strongly disagree, as shown in Figure 1.

Student ratings for each specific review standard were high, with 75% to 91% agreeing or strongly agreeing that the course reflected each best practice for 19 out of 21 standards assessed. The two standards that did not reach 75% or higher agreement were “clearly explained the use of learning materials” (72%) and “provided grading criteria tied to grading policy” (63%). The percentage of students disagreeing or strongly disagreeing that standards were met in this course was low, with all but one standard below 10% disagreement (the exception being “learning objectives were suited to the level of the course” with 11% of students disagreeing).

Students’ positive ratings were also reflected in the grand percentages calculated for general review standards in Table 1, each of which received 75% or higher agree/strongly agree ratings and disagree/strongly disagree percentages under 7%. The two highest rated general standards were Course Overview and Introduction (88% strongly agree or agree), and Learning Objectives (85% strongly agree or agree). The lowest-rated general standard was Assessment and Measurement (75% strongly agree or agree).

Preliminary analysis Cronbach’s alpha was used to examine the internal consistency of student responses across the survey questions (0.96) was high, which indicated stability in students’ survey responses, and therefore, instrument reliability. We could conclude that the instrument used to collect student’s perception of this online course is reliable.

**Figure 2.**
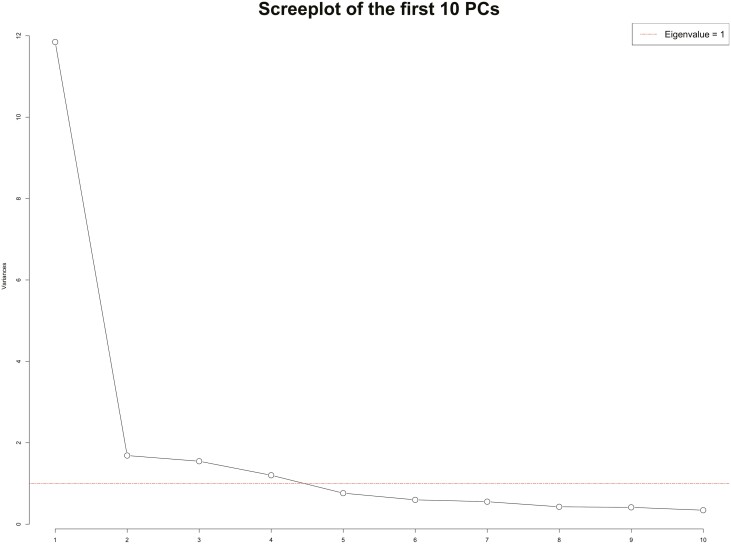
Scree plot for the first 10 PCs. The scree plot orders the eigenvalues from largest to smallest to determine how the data are spread out over the principal component. The first four principal components have eigenvalues that are greater than 1.

The results of the principal component analysis on the percentage of variation are presented in Table 2. The singular value decomposition method was used to conduct the principal component analysis. The first principal component (**PC**) explained 56.42% of the variance from student data, which accounts for the most variation in the data. About 77.53% of the data variance explained was by the first four PCs together. The scree plot shown in Figure 2 orders the eigenvalues, numbers that indicate how the data are spread out on the eigenvector (aka. principal component) from largest to smallest. In the results, the first four principal components have eigenvalues greater than 1.

**Table 2.** Eigenvalues, variance explained, and cumulative variance for survey response

PCs	Eigenvalue	Variance explained (%)	Cumulative variance (%)
1	3.44	56.42	56.42
2	1.29	8.02	64.44
3	1.24	7.36	71.81
4	1.09	5.72	77.53

In order to explore the magnitude and direction of each PC to the original QM questions, we examined the absolute values of the loading scores. The larger the absolute value of the loading, the more important the corresponding question is in calculating the PCs. Table 3 presents the first four PCs and the top 10 absolute loading scores. The first principal component has large values associated with QM standard 2, 4, and 6, especially focus on learner’s perspectives and objectives from standard 2, so this component primarily measures the Learning Objectives. The second component has large values associated with standard 5, 3, and 8, especially toward learning activities and classroom responses from standard 5, so this component primarily measures the Learner Interaction and Engagement. The third component has large values associated with standard 3, 6, 7, 8, especially focus on information about accessibility and clear technical support from 7 and 8, so this component primarily measures Accessibility and Usability. The fourth component has large values associated with standard 2, 3, and 5, especially on the clearly stated learning objectives and engagement of standard 2, so this component primarily measures Clarity.

**Table 3.** PCs and absolute loadings

Factors	Question	Absolute loadings	Factors	Question	Absolute loadings
1 Learning objectives	Q16	0.254	3 Accessibility and usability	Q21	0.423
	Q12	0.239		Q18	0.359
	Q2	0.234		Q13	0.309
	Q11	0.234		Q9	0.303
	Q19	0.233		Q19	0.298
	Q6	0.231		Q17	0.257
	Q5	0.229		Q10	0.206
	Q3	0.228		Q16	0.205
	Q17	0.225		Q20	0.19
	Q7	0.223		Q1	0.18
2 Learner interaction and engagement	Q14	0.391	4 Clarity	Q14	0.387
	Q8	0.385		Q6	0.363
	Q15	0.311		Q17	0.32
	Q1	0.308		Q1	0.286
	Q4	0.295		Q9	0.28
	Q21	0.266		Q5	0.271
	Q20	0.251		Q4	0.266
	Q10	0.247		Q7	0.263
	Q11	0.241		Q10	0.221
	Q13	0.19		Q15	0.214

## Discussion

Student interest in online and hybrid learning in higher education is growing, and that trend is expected to continue in the coming years (Garrett, Simunich, Legon, & Fredericksen, 2022). With the movement toward universal adoption of this instructional modality comes increased scrutiny on its acceptability and impact. Most higher education institutions have adopted quality assurance standards for their online and hybrid courses (Garrett et al., 2022), investing significant time and resources into learning and implementing best practices in the design of online courses. To date, however, research on student perceptions of courses that meet these standards is limited. The purpose of the current study was to determine whether students perceive a QM-certified course to be of high quality, and if so, to provide support for quality assurance efforts in online education.

The hybrid (taught partially in-person and partially online) course examined in this study was certified by QM. At the end of the course, students provided ratings for how well the course reflected each of the essential review standards. For almost all of the essential specific standards, three-quarters or more of the students agreed or strongly agreed that the course reflected the best practice described in the standard. We also examined student responses by the eight general standards in the rubric, which are overarching concepts that include several specific standards. When the data were examined in this way, we determined that their highest ratings were for the general standards that covered course overview and introduction and learning objectives. The course overview and introduction are a vital component, as it sets the tone for student engagement throughout the course. The more engaged students are, the more they are satisfied with the course and their subsequent learning (Baloran et al. 2021). Learning objectives that are specific, measurable, and clear are also fundamental, because they lead to higher learner performance (Darabi et al., 2013).

We also conducted a principal components analysis to determine meaningful clusters of standards based on our data. This method creates applicable categories for factors no matter what specific standard they fall under, as recommended by Shattuck (2015). The resulting four components were learning objectives, learner interaction and engagement, accessibility and usability, and clarity. With this knowledge, the four components can be used to positively increase student perceptions, especially in areas where they are lacking.

The results of this study demonstrate that the high quality of a QM-certified course is clear to students. The findings provide justification for the time and resource investment required to design courses that meet these standards. Student perception is an important factor in the assessment of the impact of quality assurance efforts. In our future research, we plan to build on these findings by investigating how ratings of course quality change before and after a course is modified to meet quality assurance standards, the impact of such a change on student outcomes such as retention and grades, and the influence of specific course design factors on those outcomes.

## Supplementary Material

txad112_suppl_Supplementary_Appendix

## References

[CIT0001] Bali, S., and Liu, M. 2018. Students’ perceptions toward online learning and face-to-face learning courses. J. Phys. Conf. Ser. 1108. doi:10.1088/1742-6596/1108/1/012094

[CIT0002] Baloran, E. T., J. T. Hernan, and J. S. Taoy. 2021. Course satisfaction and student engagement in online learning amid covid-19 pandemic: a structural equation model. Turk. Online J. Distance Educ. 22:1–12. doi:10.17718/tojde.1002721

[CIT0003] Darabi, A., X. Liang, R. Suryavanshi, and H. Yurekli. 2013. Effectiveness of online discussion strategies: a meta-analysis. Am. J. Distance Educ. 27:228–241. doi:10.1080/08923647.2013.837651

[CIT0004] Dixson, M. D. 2015. Measuring student engagement in the online course: the online student engagement scale (OSE). Online Learn. 19. doi:10.24059/olj.v19i4.561

[CIT0005] Garrett, R., B. Simunich, R. Legon, and E. E. Fredericksen. 2022. CHLOE 7: Tracking Online Learning from Mainstream Acceptance to Universal Adoption, The Changing Landscape of Online Education, 2022. Retrieved from the Quality Matters: https://www.qualitymatters.org/qa-resources/resource-center/articles-resources/CHLOE-7-report-2022

[CIT0006] Guppy, N., D. Verpoorten, D. Boud, L. Lin, J. Tai, and S. Bartolic. 2022. The post-COVID-19 future of digital learning in higher education: Views from educators, students, and other professionals in six countries. Br. J. Educ. Technol. 53:1750–1765. doi:10.1111/bjet.13212 https://doi-org.prox.lib.ncsu.edu/10.1111/bjet.13212

[CIT0007] He, W., G. Xu, and S. E. Kruck. 2014. Online IS education for the 21st century. J. Inform. Syst. Educ. 25:101–106. https://aisel.aisnet.org/jise/vol25/iss2/1

[CIT0008] Kaiser, H. F. 1960. The application of electronic computers to factor analysis. Educ. Psychol. Meas. 20:141–151. doi:10.1177/001316446002000116

[CIT0011] Nassoura, A. B. 2020. Measuring students’ perceptions of online learning in higher education. Int. J. Sci. Technol. Res. 9:1965–1970.

[CIT0012] QM Higher Education Rubric, Fifth Edition. 2018. Quality Matters. Used under license. Retrieved from MyQM.

[CIT0017] R Core Team (2019) R: A Language and Environment for Statistical Computing. R Foundation for Statistical Computing, Vienna, Austria. https://www.R-project.org/

[CIT0013] Sadaf, A., F. Martin, and L. Ahlgrim-Delzell. 2019. Student perceptions of the impact of “Quality matters” certified online courses on their learning and engagement. Online Learn. 23:214–233. doi:10.24059/olj.v23i4.2009

[CIT0014] Shattuck, K. 2015. Research inputs and outputs of Quality Matters: Update to 2012 and 2014 versions of What we’re learning from QM-focused research. Annapolis, MD: Quality Matters.

[CIT0015] Shattuck, K., W. Zimmerman, and D. Adair. 2014. Continuous improvement of the QM rubric and review processes: Scholarship of integration and application. Internet Learn. 3:25–34. doi:10.18278/il.3.1.3.

