# Supplementary material for: Student perceptions of the impact of quality matters essential standards in an animal physiology course
Source: Transl Anim Sci. 2023 Sep 21;7(1):txad112. doi: 10.1093/tas/txad112 (PMC10578200; doi:10.1093/tas/txad112)
Supplement: txad112_suppl_Supplementary_Appendix [file txad112_suppl_supplementary_appendix.docx]

*Appendix A*

**Student Perceptions of the Impact of QM Essential Standards in an Animal Physiology Course**

**Survey Flow**

**Appendix A**

**Consent Form (1 Question)**

**Appendix B**

**Standard: Course Overview and Introduction (2 Questions)**

**Standard: Learning Objectives (5 Questions)**

**Standard: Assessment and Measurement  (3 Questions)**

**Standard: Instructional Materials (2 Questions)**

**Standard: Learner Interaction and Engagement  (3 Questions)**

**Standard: Course Technology (2 Questions)**

**Standard: Learner Support (2 Questions)**

**Standard: Accessibility and Usability (2 Questions)**

**Appendix C**

**Demographics (4 Questions)**

**Prize Raffle Drawing Link (1 Question)**

| Page Break |  |
| --- | --- |

**Start of Block: Consent Form**

Q24 Welcome! 
You are being asked to complete a survey for research purposes. The survey is about understanding the impact of course quality improvements made to the physiology course. Completing this survey is voluntary and you can stop at any time by closing the web browser that you open the survey through. 


You must be 18 years of age or older, reside in the United States, and a student in ANS 205 taught by Dr. Shweta Trivedi at North Carolina State University to participate in this study. You do not have to be a pre-veterinary student to participate. 


The survey will take you between 8-10 minutes to complete and you will be asked questions about the learning goals, activities, and assessments, as well as how accessible the course is. You will also be asked a few demographic questions about yourself.


There are minimal risks associated with your participation in this survey. It is not a condition nor expectation of your enrollment in this course or at NC State to participate in this research. At the end of the research survey, there will be a link to a separate survey to collect your name, and email if you want to be placed in a drawing to win an NCSU shirt, tumbler, or a backpack. Only completed surveys will be entered into the drawing. Winners will be notified via email and will have one week to pick up their prize. If winners do not pick up their prize within one week, then they will forfeit their compensation. 


All survey responses will be confidential. The course professor will not seek to re-identify anyone from the surveys that are submitted. To ensure that your answers remain confidential, please take the survey in a private location with your browser in private/incognito mode.


If you have any questions about the survey itself, how it is implemented, or survey compensation, please contact Dr. Shweta Trivedi at strived@ncsu.edu or 919-515- 0266. 


If you have questions about your rights as a participant or are concerned with your treatment throughout the research process, please contact the NC State University IRB Director at IRB-Director@ncsu.edu, 919-515-8754, or fill out their confidential form online. 


Please note that this survey will be best displayed on a laptop or desktop computer. Some features may be less compatible for use on a mobile device.

- I wish to participate (1)
- I do not wish to participate (2)

**End of Block: Consent Form**

**Start of Block: Course Overview and Introduction**

*Display This Question:*

*If Welcome!  You are being asked to complete a survey for research purposes. The survey is about und... = I wish to participate*

Q1 Instructions make clear how to get started and where to find course components

- 0 - Strongly Disagree (1)
- 1 - Disagree (2)
- 2 - Neutral (3)
- 3 - Agree (4)
- 4 - Strongly Agree (5)

*Display This Question:*

*If Welcome!  You are being asked to complete a survey for research purposes. The survey is about und... = I wish to participate*

Q2 Learners are introduced to the purpose and structure of the course

- 0 - Strongly Disagree (1)
- 1 - Disagree (2)
- 2 - Neutral (3)
- 3 - Agree (4)
- 4 - Strongly Agree (5)

**End of Block: Course Overview and Introduction**

**Start of Block: Learning Objectives**

*Display This Question:*

*If Welcome!  You are being asked to complete a survey for research purposes. The survey is about und... = I wish to participate*

Q3 The course learning objectives describe outcomes that are measurable

- 0 - Strongly Disagree (1)
- 1 - Disagree (2)
- 2 - Neutral (3)
- 3 - Agree (4)
- 4 - Strongly Agree (5)

*Display This Question:*

*If Welcome!  You are being asked to complete a survey for research purposes. The survey is about und... = I wish to participate*

Q4 The module learning objectives describe outcomes that are measurable and consistent with course-level objectives

- 0 - Strongly Disagree (1)
- 1 - Disagree (2)
- 2 - Neutral (3)
- 3 - Agree (4)
- 4 - Strongly Agree (5)

*Display This Question:*

*If Welcome!  You are being asked to complete a survey for research purposes. The survey is about und... = I wish to participate*

Q5 All learning objectives are stated clearly and written from the learner's perspective

- 0 - Strongly Disagree (1)
- 1 - Disagree (2)
- 2 - Neutral (3)
- 3 - Agree (4)
- 4 - Strongly Agree (5)

*Display This Question:*

*If Welcome!  You are being asked to complete a survey for research purposes. The survey is about und... = I wish to participate*

Q6 The relationship between the learning objectives and the course activities is clearly stated

- 0 - Strongly Disagree (1)
- 1 - Disagree (2)
- 2 - Neutral (3)
- 3 - Agree (4)
- 4 - Strongly Agree (5)

*Display This Question:*

*If Welcome!  You are being asked to complete a survey for research purposes. The survey is about und... = I wish to participate*

Q7 The learning objectives are suited to the level of the course

- 0 - Strongly Disagree (1)
- 1 - Disagree (2)
- 2 - Neutral (3)
- 3 - Agree (4)
- 4 - Strongly Agree (5)

**End of Block: Learning Objectives**

**Start of Block: Assessment and Measurement**

*Display This Question:*

*If Welcome!  You are being asked to complete a survey for research purposes. The survey is about und... = I wish to participate*

Q8 The assessments measure the stated learning objectives

- 0 - Strongly Disagree (1)
- 1 - Strongly Agree (2)
- 2 - Neutral (3)
- 3 - Agree (4)
- 4 - Strongly Agree (5)

*Display This Question:*

*If Welcome!  You are being asked to complete a survey for research purposes. The survey is about und... = I wish to participate*

Q9 The course grading policy is stated clearly

- 0 - Strongly Disagree (1)
- 1 - Disagree (2)
- 2 - Neutral (3)
- 3 - Agree (4)
- 4 - Strongly Agree (5)

*Display This Question:*

*If Welcome!  You are being asked to complete a survey for research purposes. The survey is about und... = I wish to participate*

Q10 Specific and descriptive criteria are provided for the evaluation of learners' work and are tied to the course grading policy

- 0 - Strongly Disagree (1)
- 1 - Disagree (2)
- 2 - Neutral (3)
- 3 - Agree (4)
- 4 - Strongly Agree (5)

**End of Block: Assessment and Measurement**

**Start of Block: Instructional Materials**

*Display This Question:*

*If Welcome!  You are being asked to complete a survey for research purposes. The survey is about und... = I wish to participate*

Q11 The instructional materials contribute to the achievement of the stated course and module learning objectives

- 0 - Strongly Disagree (1)
- 1 - Disagree (2)
- 2 - Neutral (3)
- 3 - Agree (4)
- 4 - Strongly Agree (5)

*Display This Question:*

*If Welcome!  You are being asked to complete a survey for research purposes. The survey is about und... = I wish to participate*

Q12 Both the purpose of the instructional material and how the materials are to be used for the learning activities are clearly explained

- 0 - Strongly Disagree (1)
- 1 - Disagree (2)
- 2 - Neutral (3)
- 3 - Agree (4)
- 4 - Strongly Agree (5)

**End of Block: Instructional Materials**

**Start of Block: Learner Interaction and Engagement**

*Display This Question:*

*If Welcome!  You are being asked to complete a survey for research purposes. The survey is about und... = I wish to participate*

Q13 The learning activities promote the achievement of the stated learning objectives

- 0 - Strongly Disagree (1)
- 1 - Disagree (2)
- 2 - Neutral (3)
- 3 - Agree (4)
- 4 - Strongly Agree (5)

*Display This Question:*

*If Welcome!  You are being asked to complete a survey for research purposes. The survey is about und... = I wish to participate*

Q14 Learning activities provide opportunities for interaction that support active learning

- 0 - Strongly Disagree (1)
- 1 - Disagree (2)
- 2 - Neutral (3)
- 3 - Agree (4)
- 4 - Strongly Agree (5)

*Display This Question:*

*If Welcome!  You are being asked to complete a survey for research purposes. The survey is about und... = I wish to participate*

Q15 The instructor's plan for classroom response time and feedback on assignments is clearly stated

- 0 - Strongly Disagree (1)
- 1 - Disagree (2)
- 2 - Neutral (3)
- 3 - Agree (4)
- 4 - Strongly Agree (5)

**End of Block: Learner Interaction and Engagement**

**Start of Block: Course Technology**

*Display This Question:*

*If Welcome!  You are being asked to complete a survey for research purposes. The survey is about und... = I wish to participate*

Q16 The tools used in the course support the learning objectives

- 0 - Strongly Disagree (1)
- 1 - Disagree (2)
- 2 - Neutral (3)
- 3 - Agree (4)
- 4 - Strongly Agree (5)

*Display This Question:*

*If Welcome!  You are being asked to complete a survey for research purposes. The survey is about und... = I wish to participate*

Q17 Course tools promote learning engagement and active learning

- 0 - Strongly Disagree (1)
- 1 - Disagree (2)
- 2 - Neutral (3)
- 3 - Agree (4)
- 4 - Strongly Agree (5)

**End of Block: Course Technology**

**Start of Block: Learner Support**

*Display This Question:*

*If Welcome!  You are being asked to complete a survey for research purposes. The survey is about und... = I wish to participate*

Q18 The course instructions articulate or link to a clear description of the technical support offered and how to obtain it

- 0 - Strongly Disagree (1)
- 1 - Disagree (2)
- 2 - Neutral (3)
- 3 - Agree (4)
- 4 - Strongly Agree (5)

*Display This Question:*

*If Welcome!  You are being asked to complete a survey for research purposes. The survey is about und... = I wish to participate*

Q19 The course instructions articulate or link to the institution's accessibility policies and services

- 0 - Strongly Disagree (1)
- 1 - Disagree (2)
- 2 - Neutral (3)
- 3 - Agree (4)
- 4 - Strongly Agree (5)

**End of Block: Learner Support**

**Start of Block: Accessibility and Usability**

*Display This Question:*

*If Welcome!  You are being asked to complete a survey for research purposes. The survey is about und... = I wish to participate*

Q20 Course navigation facilitates ease of use

- 0 - Strongly Disagree (1)
- 1 - Disagree (2)
- 2 - Neutral (3)
- 3 - Agree (4)
- 4 - Strongly Agree (5)

*Display This Question:*

*If Welcome!  You are being asked to complete a survey for research purposes. The survey is about und... = I wish to participate*

Q21 Information is provided about the accessibility of all technologies required in the course

- 0 - Strongly Disagree (1)
- 1 - Disagree (2)
- 2 - Neutral (3)
- 3 - Agree (4)
- 4 - Strongly Agree (5)

*Display This Question:*

*If Welcome!  You are being asked to complete a survey for research purposes. The survey is about und... = I wish to participate*

Q22 Which gender do you identify as?

- Male/Masculine (1)
- Female/Feminine (2)
- Non-binary (3)
- Gender-Fluid (4)
- Prefer not to answer (5)

*Display This Question:*

*If Welcome!  You are being asked to complete a survey for research purposes. The survey is about und... = I wish to participate*

Q23 What is your major?

- Animal Science (1)
- Biochemistry (2)
- Biology (3)
- Genetics (4)
- Poultry Science (5)
- Zoology (6)
- Other (7) ________________________________________________

*Display This Question:*

*If Welcome!  You are being asked to complete a survey for research purposes. The survey is about und... = I wish to participate*

Q24 What is your academic status?

- Freshman (1)
- Sophomore (2)
- Junior (3)
- Senior (4)
- Non-degree Seeking (5)
- Graduate (6)

*Display This Question:*

*If Welcome!  You are being asked to complete a survey for research purposes. The survey is about und... = I wish to participate*

Q25 Are you a transfer student?

- Yes (1)
- No (2)

**End of Block: Accessibility and Usability**

**Start of Block: Prize Raffle Drawing Link**

*Display This Question:*

*If Welcome!  You are being asked to complete a survey for research purposes. The survey is about und... = I wish to participate*

Q26 If you would like to register for the prize raffle, please click the link below to be taken to a separate survey that will only ask for your name and NCSU email. 


https://ncsu.qualtrics.com/jfe/form/SV_daMwf963icNxygS

**End of Block: Prize Raffle Drawing Link**
